# In search of a recognition memory engram

**DOI:** 10.1016/j.neubiorev.2014.09.016

**Published:** 2015-03

**Authors:** M.W. Brown, P.J. Banks

**Affiliations:** University of Bristol, School of Physiology and Pharmacology, Medical Sciences Building, Bristol BS8 1TD, UK

**Keywords:** Perirhinal cortex, Familiarity, LTP, LTD, Imprinting

## Abstract

A large body of data from human and animal studies using psychological, recording, imaging, and lesion techniques indicates that recognition memory involves at least two separable processes: familiarity discrimination and recollection. Familiarity discrimination for individual visual stimuli seems to be effected by a system centred on the perirhinal cortex of the temporal lobe. The fundamental change that encodes prior occurrence within the perirhinal cortex is a reduction in the responses of neurones when a stimulus is repeated. Neuronal network modelling indicates that a system based on such a change in responsiveness is potentially highly efficient in information theoretic terms. A review is given of findings indicating that perirhinal cortex acts as a storage site for recognition memory of objects and that such storage depends upon processes producing synaptic weakening.

## Highlights

• The role of the perirhinal cortex in familiarity discrimination is reviewed.
• Behavioural, pharmacological and electrophysiological evidence is considered.
• The cortex is found to be essential for memory acquisition, retrieval and storage.
• The evidence indicates that perirhinal synaptic weakening is critically involved.

## 1 Introduction

Gabriel Horn, my long-term mentor, was an inspiration, both personally and through his research, to me and many others. His originality and clarity of thought made possible major, path-finding advances in research into the neural basis of memory. This review will make brief mention of parallels in approach between Gabriel's work in search of the memory engram underlying imprinting in the chick while describing research into the role of perirhinal cortex as a storage site for recognition memory.

A large body of data from human and animal studies using psychological, recording, imaging, and lesion techniques indicates that recognition memory involves at least two separable processes, familiarity discrimination and recollection, and that perirhinal cortex is particularly concerned with familiarity discrimination for individual items, although some disagreement remains (see for reviews and recent work: Brown and Aggleton, 2001; Brown et al., 2010; Clark and Squire, 2010, 2013; Dede et al., 2014; Eichenbaum et al., 2007; Guderian et al., 2011; Kim et al., 2014; Lech and Suchan, 2013; Martin et al., 2013; McNulty et al., 2012; Miyamoto et al., 2014; Ranganath and Ritchey, 2012; Wang et al., 2013; Warburton and Brown, 2010; Watson and Lee, 2013; Westerberg et al., 2013; Winters et al., 2008). There is wide agreement (see above references) that more complex aspects of recognition memory including recollective, contextual, associative and spatial aspects of recognition memory rely on the hippocampus. This review will focus on the familiarity discrimination component of recognition memory (has an item been experienced previously or not?) and what is known of neural changes in the perirhinal cortex of the temporal lobe that have been associated with such learning. Analysis of potential neural changes underlying the learning has mainly been carried out in rodents. Fortunately, there is good evidence to indicate that similar brain regions and potentially similar mechanisms are found also in primates (as discussed further below).

Recognition memory requires judgements concerning the physical characteristics of a stimulus (or event) and the prior occurrence of that stimulus (event). Although recognition memory relies on a stimulus being identifiable (at the least that its physical characteristics can be perceived), judgements of prior occurrence themselves do not necessarily involve the new learning of stimulus characteristics. Judgements of prior occurrence can be made for a stimulus whose physical characteristics (identity) have already been learned (‘when did you last see your father?’). Similarly, judgements of stimulus identity may be made without requiring consideration of prior occurrence (‘which of these two stimuli in front of you is a dog and which a cat?’). Accordingly, judgements of stimulus identity and of prior occurrence are potentially separable processes, although they are strongly interlinked. Manipulating the confusability of stimuli and the length of time between their appearances will alter the difficulty of both identity and recognition memory judgements. Mechanisms underlying judgement of prior occurrence are most readily studied when perceptual discrimination is made easy.

Imprinting relies on a bird recognising a specific stimulus (in nature, the mother hen). However, although the learning necessarily implies a record within the brain of the bird's prior experience of the imprinted stimulus, what is standardly measured in the laboratory is the bird's discrimination of the highly familiar imprinted stimulus from another, less familiar (‘novel’) stimulus. As the imprinted and alternative stimuli are repeatedly presented, the discrimination between them is likely to rely more on the learned physical characteristics of the imprinted stimulus (i.e. its identity) than its relative familiarity. Correspondingly, the observed brain changes produced by imprinting are likely mainly to concern learning to recognise the physical characteristics of the stimulus (together with its social/emotional import), i.e. the ability to identify the imprinted stimulus, rather than when it was last seen.

The research to be reported involving perirhinal cortex is concerned with the learning underlying judgements of the prior occurrence of a stimulus, chiefly its relative familiarity, rather than learning to identify that stimulus. *Relative* familiarity is a more conservative term than absolute familiarity as any given stimulus typically shares features with other, previously experienced stimuli so that judgements are more probably of relative unfamiliarity rather than absolute novelty. Notably, the memory to be considered is dependent upon single rather than multiple exposure learning. In the case of perirhinal cortex, the potential separation of learning types is an important issue because perirhinal cortex appears to be involved in both types of learning: multi-exposure perceptual and single-exposure prior occurrence (Bartko et al., 2007a,b, 2010; Brown and Aggleton, 2001; Buckley and Gaffan, 1998, 2006; Bussey and Saksida, 2002, 2005; Bussey et al., 2002; Clark and Squire, 2010; Eichenbaum et al., 2007; Guderian et al., 2011; Murray and Bussey, 1999a; Murray et al., 2007; Norman and Eacott, 2004; Warburton and Brown, 2010; Winters et al., 2008). If familiarity judgements are to be studied, it is important that the stimuli to be discriminated are seen infrequently – otherwise the subject is more likely to rely on information concerning recency or context in making decisions. In animal research on familiarity discrimination a ‘familiar’ stimulus may have been encountered only once previously and a ‘novel’ stimulus is likely never to have been experienced previously, and certainly not at all recently. It should be noted that this usage differs from much research with human subjects where the items presented (e.g. words or pictures of common objects) are often familiar (although unlikely to have been encountered recently).

## 2 Localising the engram

Gabriel's early work on imprinting was aimed at finding where in the chick brain learning-related changes occurred. Autoradiography was used to detect biochemical changes indicating brain regions where learning was occurring during imprinting (Bateson et al., 1973; Horn et al., 1971; Rose et al., 1970); this was then followed up with autoradiographic imaging (Horn et al., 1979). In the case of familiarity discrimination, the initial localisation of the critical region was a result rather of serendipity than a systematic approach (Brown et al., 1987). However, subsequent research used immunohistochemical imaging to identify regions showing familiarity-related changes. Such work has recently been reviewed (Aggleton et al., 2012); more recent papers include (Barbosa et al., 2013; Beer et al., 2013).

The central difficulty in localising an engram is that of separating incidental and non-specific changes from those that encode the memory itself. Many changes occur in the brain when something is learned; only a few of these changes are specific to registration of the particular memory itself. For the chick considerable ingenuity was engaged in a series of experiments that isolated changes exclusively related to learning from those arising from sensory stimulation, behavioural (motor) output, motivation, or changes in emotional or endocrinological state, or in alertness and attention (Bateson et al., 1973; Horn, 1985; Horn et al., 1971).

**Fig. 1:**
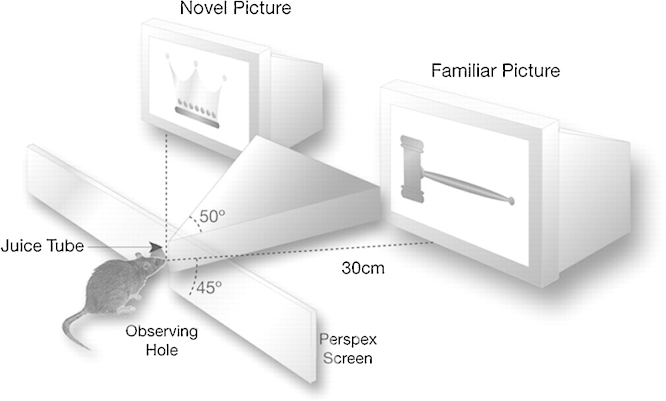
Schematic diagram of the paired-viewing apparatus. When the rat's snout is engaged in the observing hole in the Perspex screen, the right eye can see only the right monitor and the left eye can see only the left monitor. Stimuli are presented on both monitors simultaneously. Juice is delivered towards the end of picture presentation through a tube that the rat can just reach to lick when its snout is in the observing hole.

Modified with permission from Fig. 1, Brown and Aggleton (2001).

A variety of techniques have been used to provide similar exclusions in the case of recognition memory. One such is the paired viewing procedure (Zhu et al., 1996). A rat is taught that it can receive juice by maintaining its snout in a hole. While in this position the rat is shown successively a series of pairs of objects (early experiments) or pictures on a computer screen (later experiments), with one of each pair being visible only by the left eye, the other only by the right. Juice is delivered just before the pictures disappear. Over several days the rat is acclimatised to the procedure and a particular series of pictures is shown repeatedly with the intention of making them familiar. The rat also sees novel pictures, so that it becomes used to seeing both a novel and familiar picture at the same time. At test on the final day, the familiar set of pictures is displayed to one eye while simultaneously the other eye sees a series of novel pictures. This task has no behavioural contingency beyond the rat maintaining its viewing position; consequently, behaviour is the same for both familiar and simultaneously displayed novel pictures, as is the rat's level of alertness, and its motivational, emotional and endocrinological states. The association of juice delivery with the pictures is important for maintaining the rat's interest in viewing the pictures, though it raises the possibility that the familiar pictures may gain a stronger association with reward than the novel. How the rat's attention might be directed during viewing is unknown. Sensory input is matched as the same number of novel and familiar pictures are seen and, across rats, the sets of novel and familiar pictures are counterbalanced so a novel picture for one rat is familiar for the next, and vice versa. Similarly, across rats the eye viewing the novel pictures is also counterbalanced. Brain activation is then compared between the two hemispheres, the rat becoming its own control. By displaying the pictures in the monocular fields of each eye, information initially passes to the opposite hemisphere, for novel pictures on one side and familiar on the other. Fortuitously, most of the information does not re-cross to the opposite hemisphere, so novel-familiar differences are indeed found without a requirement to sever the corpus callosum. It seems humans also process most of such complex visual information in one hemisphere as there is an advantage in recognition memory if familiarity judgements are made using information presented to the same hemisphere, compared to when information is first presented to one hemisphere then to the other (Hornak et al., 2002) (Fig. 1).

Across a series of experiments using the paired viewing procedure, higher numbers of activated neurons (as detected by their generation of immunohistochemically labelled Fos protein) have been found in the perirhinal cortex and adjacent visual association cortex (termed ‘Te2’) in the hemisphere processing the novel rather than the familiar individual items (see for review; Aggleton et al., 2012). No consistent changes were found in the hippocampus. Accordingly, these data provide strong evidence that cellular processes closely linked to registration of prior occurrence occur in perirhinal and adjacent cortex. Using the same procedure but with the novelty being provided by spatial rearrangements of familiar items rather than by the individual items themselves, activation differences were found in the hippocampus but not the perirhinal cortex; so the differential activation of the hippocampus and perirhinal cortex by spatial and non-spatial novel and familiar stimuli can be doubly dissociated (Wan et al., 1999). It is worth noting that the hippocampus is therefore involved in the familiarity of associations between stimuli rather than the familiarity of the stimuli themselves.

Accordingly, regions other than perirhinal cortex may also be involved in recognition memory processes depending upon the type of information and type of recognition memory task. Perirhinal cortex appears to have a special role in visual rather than auditory or haptic memory (Albasser et al., 2011a,b, 2013a,b; Fritz et al., 2005; Kowalska et al., 2001; Wan et al., 2001; Winters and Reid, 2010). Regions other than perirhinal cortex are important for recognition memory based on somatosensory (Albasser et al., 2011a,b; Winters and Reid, 2010) or auditory information (Fritz et al., 2005; Kowalska et al., 2001; Wan et al., 2001), though perirhinal cortex is necessary for olfactory recognition memory (Otto and Eichenbaum, 1992). There are also indications that the involvement of perirhinal cortex and hippocampus may differ if the task employs multiple rather than single exposure trials (Aggleton et al., 2012; Albasser et al., 2011a; Forwood et al., 2005; Gaskin et al., 2010; Mumby et al., 2005). The following sections will therefore concentrate on studies where the use of visual information concerning individual objects and single exposure learning may be expected to engage perirhinal cortex.

## 3 Establishing necessity for learning

The activation (or deactivation) of a brain region during a task does not establish that that region is necessary for the learning or memory required for task performance: the activated region may merely passively reflect activation of some other critical region or may operate as a parallel to another critical region. A specific, selective learning deficit produced by interference with the operation of a target region is required to demonstrate an essential role for that region. Even such a selective behavioural deficit does not establish that the region is a site of learning or memory because the interference may impair other processes necessary to task performance or may prevent the proper operation of some distal region that holds the engram. It needs to be shown that interference with the target region produces a selective behavioural impairment that does not depend upon general changes to sensory processing, motor abilities, or motivational/emotional or attentive/alertness states. It also needs to be shown that similar impairment is not produced by interference with other regions whose anatomical connections suggest might potentially play significant roles. Nevertheless, for both chick imprinting and for familiarity discrimination, the effects of selective lesions have established that the region identified through activation was also necessary for the learning: the intermediate medial mesopallium (IMM; previously the intermediate and medial hyperstriatum ventrale) for imprinting (Horn et al., 1983; McCabe et al., 1981) and perirhinal cortex for single item familiarity discrimination.

Ablation of the perirhinal cortex in either the rat or the monkey produces impairment of visual recognition memory tasks that rely on familiarity discrimination of single items (Brown and Aggleton, 2001; Clark and Squire, 2010; Eichenbaum et al., 2007; Guderian et al., 2011; Warburton and Brown, 2010; Winters et al., 2008). A similar impairment has also been reported for a human subject with perirhinal damage (Bowles et al., 2007) and for a group of human patients with temporal lobe epilepsy that experience déjà vu (Martin et al., 2012). Perirhinal ablation in the rat does not produce impairment of visual discrimination for the types of item used in the recognition memory tasks, and produces no effects on motor abilities, motivational/emotional or attentive/alertness states that could provide explanation of the memory deficit. However, perirhinal ablation can produce impairment in the performance of complex visual discrimination problems (Bartko et al., 2007a,b, 2010; Buckley and Gaffan, 1998, 2006; Bussey and Saksida, 2002, 2005; Bussey et al., 2002; Murray et al., 2007; Murray and Bussey, 1999b; Nadel and Peterson, 2013; Norman and Eacott, 2004; though see: Knutson et al., 2013). Small perceptual differences remain detectable after perirhinal lesions, so it is confusability or interference across stimuli that result in impairment (Eacott et al., 2001). Accordingly, when aiming to measure memory impairments produced by perirhinal lesions it is important to avoid readily confusable stimuli, minimise potential interference (Bartko et al., 2010; Bussey and Saksida, 2005), and check for potential perceptual deficits.

**Fig. 2:**
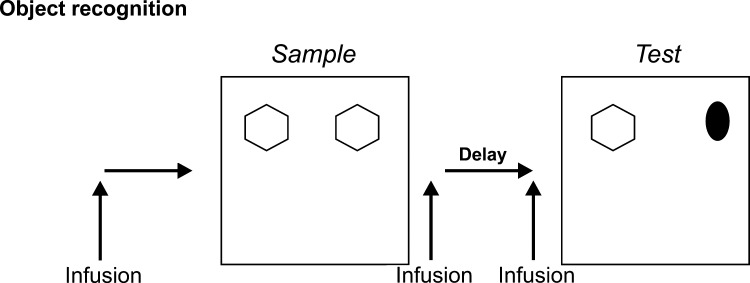
Rodent object recognition memory task. A rat is exposed to and allowed to explore two copies of an object during the sample phase. After a delay interval, the rat is allowed to explore a third copy of the object already explored in the sample phase and a novel object. Normal rats spend more time exploring the novel than the familiar object in the test phase. Infusions of drugs may be made prior to the sample phase to interfere with acquisition and early consolidation, at various times after the sample phase during the delay interval to interfere with consolidation and storage mechanisms, or prior to the test phase to interfere with retrieval mechanisms.

Most studies of rat recognition memory have made use of the rat's spontaneous preference to explore novel rather than familiar stimuli (Dix and Aggleton, 1999; Ennaceur and Delacour, 1988). For single object recognition memory, the rat explores an object at acquisition (sample phase) and after a delay is allowed to explore that object and a new object at test (choice phase). In what follows, unless otherwise indicated, results have been obtained using such a spontaneous exploration task, with exploration being in a square arena or Y-maze (Winters et al., 2004). Importantly, the version of the task in the square arena where two copies of the same object are explored during the sample phase and a third copy of the now familiar object together with a novel object explored during the choice phase (AA → AB) does not require the hippocampus (Barker and Warburton, 2011; Forwood et al., 2005; Good et al., 2007; Langston and Wood, 2010; Mumby et al., 2002; Winters et al., 2004; though see: Clark et al., 2000, 2001; Cohen et al., 2013). The task variant in which two different objects are shown in the sample phase, one being replaced in the choice phase (AB → AC) *is* affected by hippocampal lesions (Clark and Squire, 2010; Mansuy et al., 1998; Oh et al., 2010; Pittenger et al., 2002; Barker, G.R.I. unpublished observations). Accordingly, precise details of task procedures may influence whether recollective/spatial (hippocampal) or familiarity/object (perirhinal) mechanisms are engaged: there is evidence that rats can use both (Fortin et al., 2004; Sauvage et al., 2008, 2010). Results reported below are from the AA → AB, hippocampally independent task. Although spontaneous exploration tasks remove complexities introduced by associating stimuli with rewards or punishments, performance of the task is easily influenced by general motivational and emotional influences upon the attentive state of the subjects and their willingness to explore. For example, anxiety may produce neophobia (Ennaceur, 2010). It is important to check that there is no difference between experimental and control groups in total exploration times in the sample and test phases, though this still provides a coarse rather than fine-grained measure of any potential bias produced by such influences. The task exposes stimuli over a period of minutes rather than the few seconds typically employed in other recognition memory tasks (the paired viewing procedure, for example). Further, it allows engagement of the somatosensory and olfactory systems as well as the visual (Fig. 2).

In the rat, lesions of the hippocampus or medial prefrontal cortex (regions which have also been shown to be involved in recognition memory) do not produce such severe deficits in single item visual familiarity discrimination as does perirhinal ablation (Brown and Aggleton, 2001; Brown et al., 2010; Clark and Squire, 2010; Forwood et al., 2005; Mumby et al., 2007; Vann and Albasser, 2011; Warburton and Brown, 2010; Winters et al., 2004, 2008). However, lesions of the hippocampus produce severe deficits when familiarity discrimination involves spatial information (Barker and Warburton, 2011; Brown and Aggleton, 2001; Dere et al., 2006; Eichenbaum et al., 2007; Murray et al., 2007; Squire et al., 2007; Warburton and Brown, 2010; Winters et al., 2008). As spatial familiarity discrimination is intact after perirhinal lesions, the roles of perirhinal cortex and hippocampus in object and spatial familiarity discrimination may be doubly dissociated. A similar double dissociation of function between perirhinal cortex and hippocampus in recognition memory has been established using human subjects (Bowles et al., 2010; see for reviews: Brown et al., 2010; Montaldi and Mayes, 2010; Vann and Albasser, 2011; for an alternative view see: Squire and Wixted, 2011).

In the rat, Fos immunohistochemical imaging indicates that association cortex (Te2) adjacent to perirhinal cortex also demonstrates greater activation by novel than familiar items. Lesions of this cortex produce deficits in familiarity discrimination when delays are more than 15 min, and the deficit does not seem to arise from perceptual dysfunction (Ho et al., 2011). The effects of lesions at earlier stages of the visual pathway have not been studied as any apparent amnesia would be difficult to dissociate from the expected perceptual deficit. In the monkey, interference with anterior visual association cortex (anterior TE) produces perceptual impairments but familiarity discrimination deficits have not been reported (Buffalo et al., 1999, 2000; Overman et al., 1990).

It is possible to interfere with a region's function in ways other than by ablation. In the rat a large number of studies have explored the effects of substances delivered by bilateral infusions within perirhinal cortex. The region targeted is perirhinal cortex as defined by Shi and Cassell (1999), elsewhere termed posterior perirhinal cortex (Albasser et al., 2009; Burwell, 2001). Conveniently, a single infusion can reach nearly all this region with minimal spread to surrounding areas (Attwell et al., 2001; Izquierdo et al., 2000; Martin, 1991; Seoane et al., 2009). Such infusions require controls for effects produced by the infusion process itself, including any by the vehicle used to deliver the active compound. Commonly, therefore, vehicle infusion is used as a control. Using localised drug delivery has major advantages over systemic delivery where a drug potentially affects the whole of the animal, including the whole of the brain; side-effects are minimised and drugs that cannot be delivered systemically can be used. A disadvantage is that the drug concentration at the target site is difficult to determine. However, for several compounds – the muscarinic cholinergic antagonist scopolamine (Warburton et al., 2003); the nicotinic antagonist MLA (Tinsley et al., 2011); the benzodiazepine lorazepam (Wan et al., 2004) and the L-type voltage dependent calcium channel blocker verapamil (Seoane et al., 2009) – the effects on memory of localised perirhinal infusions have been found to be the same as those for systemic delivery (where the drug concentration is known).

One particular advantage of drug administration over ablation is that selective effects upon acquisition, storage and retrieval processes may be studied by delivering the drug to be active during one but not another of these processes. It should be emphasised, however, that the cascade of consolidation processes necessary for storage start immediately following acquisition so that only fuzzy rather than completely clear divisions can be made between acquisition, consolidation and storage. In the rat spontaneous exploration recognition memory task the sample (‘acquisition’) phase typically lasts a few minutes to allow the rat ample time for exploration of the object. Hence even if a drug is administered only a minute or so after the sample phase, consolidation has potentially already been under way for a few minutes. Moreover, there are two further complications in making divisions between such memory processes: (i) For some types of learning ‘systems consolidation’ has been found to continue across many days (Dudai, 2012; Tayler and Wiltgen, 2013). (ii) Re-presentation of a stimulus (as during the test phase) activates ‘reconsolidation’ processes that render earlier memories labile (Dudai, 2012; Nader and Einarsson, 2010; Nader et al., 2000). Accordingly, even when a drug is infused to be active only during the retrieval phase of a task, some disruption of storage processes may be occurring. These issues will be discussed further below. By using compounds that switch off neuronal activity, a reversible lesion may be produced. By such means perirhinal cortex has been shown to be essential for acquisition, storage and retrieval processes.

The involvement of perirhinal mechanisms in storage mechanisms will be considered in a later section. Evidence concerning retrieval and acquisition processes is discussed next.

Only a few compounds have been found to affect recognition memory retrieval. Inactivation of perirhinal cortex (e.g. by CNQX which antagonises AMPA and kainate glutamate receptors and thereby blocks most excitatory transmission, or lidocaine which blocks axonal transmission) demonstrates that an active perirhinal cortex is necessary for retrieval processes (Hannesson et al., 2004a,b; Winters and Bussey, 2005a,b). Retrieval is also impaired by the L-type voltage-dependent calcium channel blocker verapamil (Seoane et al., 2009).

In contrast to retrieval mechanisms, rat recognition memory acquisition is far more readily disrupted. Acquisition (or early consolidation) is impaired by infusing perirhinal cortex with antagonists of NMDA (Barker et al., 2006b; Winters and Bussey, 2005a; though see: Abe et al., 2004), kainate (Barker et al., 2006b), and metabotropic glutamate receptors (Barker et al., 2006a), muscarinic and nicotinic cholinergic receptors (Bartko et al., 2014; Tinsley et al., 2011; Warburton et al., 2003; Winters et al., 2006; though see: Abe et al., 2004), dopamine D1 receptors (Balderas et al., 2013a), the benzodiazepine lorazepam (Wan et al., 2004) and L-type voltage-dependent channel blockers such as verapamil (Seoane et al., 2009). Hence multiple types of glutamatergic and cholinergic neurotransmitter receptors within perirhinal cortex are involved in encoding the occurrence of stimuli.

A surprising finding for both cholinergic and glutamatergic neurotransmission was that antagonism of different types of receptors produced different temporal patterns of amnesia (Abe and Iwasaki, 2001; Barker et al., 2006b; Barker and Warburton, 2008; Tinsley et al., 2011; Warburton et al., 2003; Winters and Bussey, 2005a). Familiarity discrimination at short delays (<1 h) was unimpaired by NMDA glutamate or nicotinic cholinergic antagonism, while at long delays (e.g. 24 h) memory was lost. The magnitude of the difference in memory for short and long delays makes it unlikely that relative task difficulty underlies the change. More probably the shorter-term memory is either held in a different region unaffected by the perirhinal infusions or by a mechanism within perirhinal cortex that is independent of NMDA and nicotinic receptor activation. Perirhinal infusions of kainate glutamatergic and muscarinic cholinergic antagonists established that the latter possibility is correct. Kainate or muscarinic antagonism within perirhinal cortex resulted in a most unusual pattern of amnesia where familiarity discrimination was impaired at short delays (<1 h) but memory was normal at longer delays (e.g. 24 h) (in contrast to the above, here the less difficult, shorter delay with less opportunity for interference resulted in a deficit). The sets of results hence provide functional double dissociations. Accordingly, there is no region outside perirhinal cortex that can support familiarity discrimination for single objects at short delays when perirhinal cortex is under kainate or muscarinic antagonism during acquisition. Moreover, there is also no region outside perirhinal cortex that can support familiarity discrimination for single objects at long delays when perirhinal cortex is under NMDA or nicotinic antagonism during acquisition. A normally functioning perirhinal cortex is therefore necessary for acquisition of such familiarity discrimination at either short or long delays, and no other region substitutes for its function. These findings provide a strong argument that recognition memory deficits produced by perirhinal dysfunction do not arise merely from increased interference (Bartko et al., 2010; Bussey and Saksida, 2005) but may also be produced by that other cause of forgetting, ‘temporal decay’ (here from impairment of acquisition processes, i.e. a failure of registration). Although increased interference during the longer interval may explain the deficit produced by infusion of an NMDA or nicotinic antagonist at the longer rather than the shorter delay, increased interference cannot simultaneously explain the deficit at the shorter rather than the longer interval (i.e. when there should be less interference) produced by infusion of a kainate or muscarinic antagonist (see for a fuller discussion: Banks et al., 2012). It further follows that within perirhinal cortex there are two separable acquisition mechanisms supporting familiarity discrimination: one dependent on kainate and muscarinic receptors and another dependent on NMDA and nicotinic receptors. The possibility that these two mechanisms underlie differences in the patterns of neuronal responsiveness that have been described for perirhinal cortex will be discussed in the next section.

## 4 Learning-related neuronal responses

Imprinting produces increased neuronal responsiveness to the imprinted stimulus in IMM (Brown and Horn, 1994; Nicol et al., 1995). Surprisingly, when the responses of individual neurons are tracked across training (stimulus exposure) sessions their responses do not increase monotonically (Horn et al., 2001). Indeed, a period of sleep soon after training is necessary if increased neuronal responsiveness and imprinted behaviour is to be maintained in the longer-term (Jackson et al., 2008). Nevertheless, the selective increase in responsiveness to the training stimulus for neurons in the region necessary for imprinted behaviour provides a potential basis for explaining that behaviour.

Neurons in monkey perirhinal cortex show response changes that signal information of potential use to recognition memory (see for review: Brown and Xiang, 1998): the region necessary for familiarity discrimination contains neurons whose responses provide a potential basis for explaining how such discrimination might be made. Many neurons (up to 25% of recorded samples) in monkey perirhinal and adjacent inferior temporal cortex respond more strongly to novel than to familiar visual stimuli (Brown et al., 1987; Eskandar et al., 1992; Fahy et al., 1993; Li et al., 1993; Miller and Desimone, 1994; Miller et al., 1993; Riches et al., 1991; Sobotka and Ringo, 1996; Xiang and Brown, 1998; see for reviews: Brown et al., 2010; Brown and Xiang, 1998; Desimone, 1996; Ringo, 1996); though in one report (Thome et al., 2012) such responses were not found when monkeys passively viewed many stimuli where the familiarity of the stimuli was irrelevant to reward. Familiarity response changes cannot be explained by changes in behavioural output (motor responses; including eye movements and pupillary changes), motivational/emotional/endocrinological factors, or changes in alertness and attention (Brown and Xiang, 1998; Desimone, 1996; Li et al., 1993; Miller and Desimone, 1994; Miller et al., 1993; Xiang and Brown, 1998). The earliest signal of a difference in neuronal response between novel and familiar stimuli in perirhinal and adjacent visual association cortex (anterior TE) occurs at ∼75 ms (±15 ms). This is little different from the shortest latencies of neuronal response to visual stimulation found in that cortex. Thus certain neurons in this cortex signal whether a stimulus has been seen before or not about as fast as this cortex signals the presence of a visual stimulus. Such early signals of relative familiarity occur before pupillary or eye movement changes (indeed, any such eventual ocular changes presumably result from output from this cortex). The speed of the initial relative familiarity signal rules out any possibility that this initial response results from feedback from non-adjacent regions such as the hippocampus or prefrontal cortex (however, it does not exclude such potential influence for later parts of the response).

In addition, the familiarity-related response changes found in perirhinal cortex and anterior TE are not explicable as passive reflections of changes earlier in the visual pathway. Differences in response between novel and familiar stimuli occur in more posterior visual cortical areas but such differences do not last across long time intervals (<few seconds) and are disrupted by presentation of other stimuli (see for review: Brown and Xiang, 1998). The familiarity-related response changes found in perirhinal cortex and anterior TE typically persist across long delays (in many cases at least 24 h) and the presentation of many interfering presentations of other stimuli (Brown and Xiang, 1998). Accordingly, these response differences evidence access to information held in long-term memory. Hence the familiarity-related response changes of perirhinal neurons must be generated within perirhinal cortex or its neighbouring visual association cortex (anterior TE): the changes are neither fed forward nor fed back from other regions.

If perirhinal neuronal responses are sought when familiar as well as unfamiliar stimuli are repeated, more than one pattern of response change is uncovered (Brown and Aggleton, 2001; Fahy et al., 1993; Li et al., 1993; Miller et al., 1993; Xiang and Brown, 1998). The existence of more than one pattern of response change implies more than one underlying plasticity mechanism (Fahy et al., 1993; Xiang and Brown, 1998). For some neurons (originally termed ‘novelty’ or ‘recency’ neurons) a reduced response is found even if a novel stimulus is presented a second time <1 s later (Li et al., 1993; Miller et al., 1993): the response change is very fast. For other neurons (‘familiarity’ neurons) the response difference only develops over a period of minutes (Xiang and Brown, 1998) but then is particularly long-lasting: ‘slow-change’ neurons (Brown et al., 2010). It should be noted that when stimuli are repeated many times or recordings are made during the performance of other types of memory tasks, other types of response changes can be found in perirhinal cortex, but these do not readily provide a basis for general familiarity discrimination (see for discussion: Brown et al., 2012). In particular, when a monkey has to respond to a single previously seen target stimulus to gain reward, certain perirhinal neurons have an increased response to the second (rewarded) appearance of the target stimulus (Miller and Desimone, 1994). However, such increased responses with stimulus repetition have only been found when a single target has to be kept in mind at one time and the monkey has been trained on that specific task. It is probable that a short-term memory or attentive mechanism is responsible as such effects have not been shown using long delays or more general recognition memory tasks. Indeed, with long delays the response to the second showing of an initially unfamiliar stimulus is reduced compared to that for the initial appearance in the overwhelming number of cases; population measures of activity display significant increases, while reductions in responses to repeated stimuli occur less often than expected by chance (Brown and Xiang, 1998). Although it is possible that the tuning of some smaller population of neurons is sharpened at the same time as the activity of many is reduced, the rest of the brain seems unlikely to ignore a signal of such magnitude that it is apparent even in population measures of perirhinal activity.

**Fig. 3:**
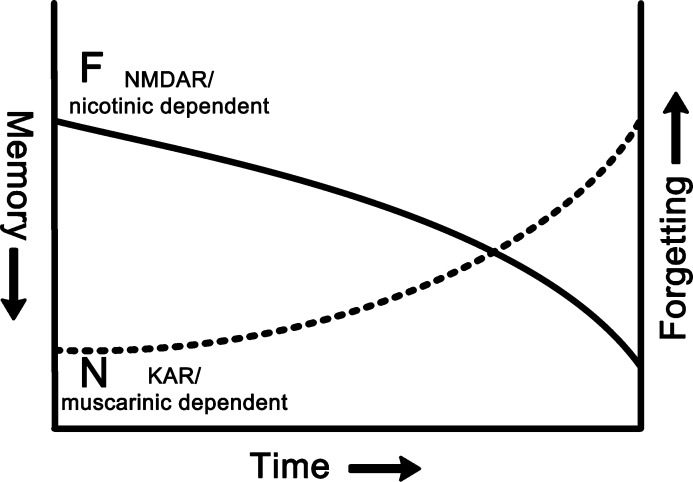
Schematic representation of memory decays (‘forgetting curves’) produced by selective cholinergic and glutamatergic antagonists. The curves are based on forgetting curves derived from population measures of monkey perirhinal neuronal responses to novel and familiar stimuli after different delays for novelty (N) and familiarity (F) neuronal types (Xiang and Brown, 1998). If kainate (KAR) and muscarinic receptor antagonists target ‘novelty’ (fast synaptic change) neurons, while NMDA and nicotinic receptor antagonists target ‘familiarity’ (slow synaptic change) neurons, then the different forgetting curves provide potential explanation for the different amnesic effects observed: NMDA or muscarinic antagonism results in short-term memory followed by forgetting at longer intervals, whereas kainate (KAR) or muscarinic antagonism produces short-term forgetting followed by long-term remembrance.

Modified with permission from Fig. 4, Brown et al. (2012).

Extrapolating across species from monkey to rat, the different temporal patterns of amnesia produced by different glutamatergic and cholinergic antagonists is potentially explicable if: (i) kainate and muscarinic antagonists interfere with plasticity mechanisms underlying the development of the fast-change responses while leaving slow-change mechanisms intact; whereas (ii) NMDA and nicotinic antagonists interfere with plasticity mechanisms underlying the development of the slow-change responses while leaving fast-change mechanisms intact (Brown et al., 2010, 2012) (Fig. 3).

Whereas there has been extensive study of perirhinal neuronal responses in the monkey (see for reviews: Brown et al., 2010; Brown and Xiang, 1998), there have been few studies in the rat. In one recent study (Deshmukh et al., 2012) responses to objects were described, but relative familiarity was not explored. In another (Burke et al., 2012), no differences in neuronal responses were found when rats ran past novel compared to familiar objects; however, neuronal activity was averaged over long time periods and the number of repetitions where objects were novel was low. In other studies (Zhu and Brown, 1995; Zhu et al., 1995), neurons with patterns of responsiveness similar to those in the monkey (‘novelty’ and ‘familiarity’ type responses) have been described when rats are shown lists of novel and familiar objects, but there has been no description of response latencies or how long the response changes persist. Accordingly, perirhinal neuronal responses have been described in the rat that are consistent with evidence for perirhinal cortex being the site of learning and storage for familiarity discrimination, but more work is required if strong support is to be provided for such a conclusion.

A parallelism of mechanisms between humans and monkeys is suggested by reductions in BOLD fMRI signals to old compared to new stimuli in the region of perirhinal cortex (Gonsalves et al., 2005; Henson et al., 2005; Montaldi et al., 2006). Moreover, MEG signal reduction to familiar compared to novel stimuli occurs in human medial temporal cortex at a latency of 85–115 ms in a rewarded recognition memory task (Bunzeck et al., 2009); this latency is little longer than that for the monkey (60–90 ms), a difference readily explained by the difference in brain size (Brown, 2009; Xiang and Brown, 1998). Hence, across species there is evidence that perirhinal cortex provides a novelty signal: its output is higher for a novel than for a familiar stimulus.

**Fig. 4:**
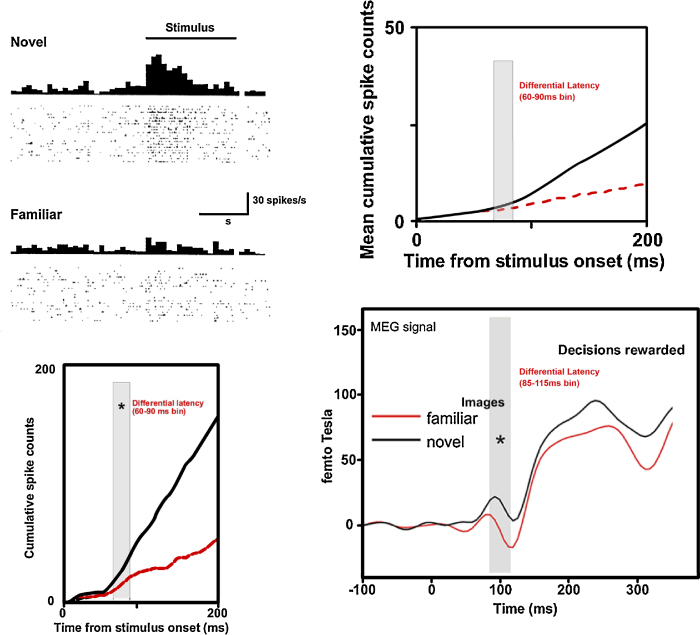
Novelty detection latency in monkey temporal cortex. *Top left panel*: Responses of a neuron to presentations of novel and familiar stimuli. Peristimulus histograms show the average firing rate for novel and for familiar stimuli. Dots beneath each histogram show the times of occurrence of individual action potentials on each trial on which a single novel or familiar stimulus was shown. *Bottom left panel*: Cumulative action potential count after stimulus onset for the novel and familiar trials. A statistically significant difference was established by the 60–90 ms time bin. *Top right panel*: Population average of such individual neuronal cumulative action potential counts for neurons whose responses change with stimulus familiarity. Novel and familiar population responses first differ significantly in the 60–90 ms time bin (Xiang and Brown, 1998) (Left two panels adapted with permission from Brown and Bashir, 2002). *Bottom right panel*: Human MEG signals for novel and familiar stimuli. There is a larger signal for novel than familiar stimuli in the 85–115 ms time bin when subjects’ responses earned reward (the asterisk denotes a significant difference). The differential latency is closely similar to that in the monkey when account is taken of the difference in monkey and human brain sizes.

Adapted with permission from Bunzeck et al. (2009).

When an initially novel stimulus is shown again, certain neuronal responses in perirhinal cortex in monkeys and rats become weaker. Such a response decrement could be produced most simply by weakening synapses that are excited by the first presentation of a novel stimulus (Brown and Xiang, 1998). Although this may seem a surprising learning mechanism, its effect is to make a network responsive to what is not shared across stimuli: the network potentially becomes a novelty detector. In contrast, enhancing synaptic strength when features are repeatedly encountered results in a network that responds strongly to features that are held in common across stimuli: the network potentially becomes a feature detector (Fig. 4).

There is a further reason for expecting the primary plasticity mechanism in perirhinal cortex to involve synaptic weakening. Computational modelling of familiarity discrimination has established that for networks where activity is not almost totally decorrelated across all component elements (a condition which would be unrealistic between neurons in the real brain), the plasticity mechanism must include the weakening of synapses (Androulidakis et al., 2008; Bogacz and Brown, 2003; Lulham et al., 2011; Norman, 2010). If the model does not include synaptic weakening, the network's storage capacity is very greatly reduced (Bogacz and Brown, 2003). Networks with synaptic weakening can achieve very high storage capacity: indeed, a network of the size of the human perirhinal cortex could potentially register and store the occurrence of a new stimulus every several seconds of a whole human life-time (Bogacz and Brown, 2003). Such models have also been shown to mimic characteristics of human familiarity discrimination (Lulham et al., 2011; Norman, 2010). Accordingly, even if sharpening of tuning of neuronal responses produced by synaptic enhancement occurs in conjunction with response reductions, synaptic weakening as a storage mechanism is essential to familiarity discrimination.

## 5 Involvement in consolidation and storage

Memory consolidation involves a cascade of processes initiated by acquisition of information and leading to its storage. Commonly, consolidation had been presumed to be complete within a few hours. However, it is now clear that processes involved in long-term memory storage are more complex. Long-term memory can be altered by sleep (e.g. Jackson et al., 2008; see for review: Stickgold, 2013). Stored (‘consolidated’) memories may be rendered labile by new encounters with aspects of an original learning experience (becoming liable to so-called ‘reconsolidation’) (Dudai, 2012; Nader and Einarsson, 2010; Nader et al., 2000). For certain types of hippocampally dependent memory ‘systems consolidation’ may continue over a period of many days or weeks, with the engagement of new storage sites (Dudai, 2012; Tayler and Wiltgen, 2013).

Studies of chick imprinting established that the left IMM is a long-term memory storage site (see for reviews: Horn, 1985, 2004) and that imprinting resulted in changes in its synaptic apposition zones (Bradley et al., 1981). Biochemical changes related to consolidation mechanisms and synaptic plasticity have also been found (Horn, 2004; Solomonia et al., 2005, 2008). In contrast, and with parallels to mammalian hippocampal learning (Dudai, 2012; Tayler and Wiltgen, 2013), the right IMM over the first few hours after training is necessary for the setting up of a further long-term store (termed S’) (Horn, 1985, 2004). Moreover, long-term memory for imprinting requires a period of sleep shortly after training (Jackson et al., 2008). In spite of the differences in brain architecture across the taxonomic classes, learning mechanisms show striking parallels.

For familiarity discrimination in the rat, no study has yet reported the effects of sleep on memory. There have been few studies of long-term systems-type consolidation for familiarity discrimination but indications are that the role of perirhinal cortex remains the same across weeks (Mumby et al., 2002). (As mentioned above, computational modelling indicates that the storage capacity of perirhinal cortex could be sufficiently high that transfer of information to another site is unnecessary.) However, some evidence of possible information transfer between perirhinal cortex and Te2 will be discussed below. ‘Reconsolidation’ effects have been reported (Balderas et al., 2013b; Silingardi et al., 2011; Winters et al., 2011) but so far not extensively studied. In contrast, there is a growing body of knowledge concerning consolidation and synaptic plasticity mechanisms in perirhinal cortex underlying familiarity discrimination and a report of perirhinal synaptic remodelling after performance of an object recognition memory task (Platano et al., 2006).

If ablation or inactivation of a region produces a memory deficit, this region is either the site of memory storage or is necessary for access to the storage site. If memory is impaired when a region is interfered with while acquisition and retrieval processes are left intact, this region is necessary for consolidation or storage of the memory. Ablation of a memory store should produce both anterograde and retrograde amnesic effects. Ablation of perirhinal cortex produces both retrograde (Mumby et al., 2002) and anterograde (Brown and Aggleton, 2001; Clark and Squire, 2010; Eichenbaum et al., 2007; Guderian et al., 2011; Warburton and Brown, 2010; Winters et al., 2008) memory impairments. The fact that retrograde amnesia was not temporally graded (Mumby et al., 2002), indicates that the role of perirhinal cortex is not temporally limited (at least up to 5 weeks) in the rat. Nevertheless, both anterograde and retrograde amnesic effects could also be produced by ablation of a region required for transmission of or access to critical information stored elsewhere. Hence ablation of perirhinal cortex establishes the necessity of the region for the task but not that the region is necessary because of its information storage. It is important to address possible transmission rather than storage of information by perirhinal cortex both because perirhinal cortex has perceptual as well as mnemonic functions (Bartko et al., 2007a,b; Buckley and Gaffan, 1998, 2006; Bussey and Saksida, 2002; Bussey et al., 2002; Murray and Bussey, 1999a; Murray et al., 2007; Murray and Wise, 2012; Norman and Eacott, 2004) and because perirhinal cortex is a major source of potential input to the hippocampal formation (Furtak et al., 2007; Witter et al., 2000).

It is therefore necessary to establish that an intervention producing memory impairment interferes with mechanisms implicated in consolidation or storage without affecting normal transmission of information. It becomes highly probable that a targeted area is a site of consolidation and storage if normal transmission remains intact because access to a storage site elsewhere should be unaffected. Presuming storage involves synaptic change, evidence of such change should be found at the putative storage site. Ideally, artificially inducing such synaptic change in the region should result in it being possible to demonstrate behavioural evidence of a memory having been implanted (see for an example involving chick imprinting (McCabe et al., 1979)).

Interfering with mechanisms involved in consolidation and synaptic plasticity within perirhinal cortex results in familiarity discrimination deficits (see below). Memory is typically tested after a delay of 24 h, with drugs being infused after acquisition (after the sample phase) and sufficiently long before retrieval (before the test phase) for them to be no longer active during retrieval. Hence during acquisition and retrieval perirhinal cortex is operating without active interference; indeed, all brain regions should be operating normally at those times. When studying consolidation mechanisms for processes underlying memory that lasts for <1 h, drugs given after acquisition are likely to remain active during retrieval, providing consequent difficulties in interpretation. Moreover, as there are known differences in the mechanisms underlying shorter and longer term memory mechanisms in perirhinal cortex (as discussed previously), conclusions drawn from impairments found after a 20 min delay may not apply to those after a 24 h delay and vice versa. Furthermore, in studies of consolidation and plasticity mechanisms it needs to be remembered that when one intracellular signalling mechanism is interfered with, many other biochemical pathways will also be affected.

Drug infusion into perirhinal cortex during the memory delay interval has established that familiarity discrimination (measured after a 24 h delay) is impaired if perirhinal transmission is silenced by infusing CNQX or lidocaine shortly, but not >40 min, after acquisition (Winters and Bussey, 2005a,b). The implication is that perirhinal activity is required during the first hour of consolidation. The critical disruption could be because of interrupted interneuronal signalling or disrupted intracellular signalling, or both. Infusing anisomycin to block protein synthesis in perirhinal cortex also impairs long-term (>90 min) recognition memory (Balderas et al., 2008). Protein synthesis has been shown to be necessary to long-term memory in many systems (see for reviews: Davis and Squire, 1984; Kandel, 2001), including chick imprinting (Takamatsu and Tsukada, 1985).

As described above, measuring Fos protein has provided a reliable marker of regions involved in recognition memory processes (see for review: Aggleton et al., 2012). An explanation for this correspondence is provided by the finding that familiarity discrimination is impaired if Fos production in perirhinal cortex is inhibited by infusing an oligodeoxynucleotide (ODN) after acquisition and during the 24 h delay period. Fos production in other systems has been linked to memory consolidation and plasticity mechanisms, particularly synaptic weakening (Herdegen and Leah, 1998; Herrera and Robertson, 1996; Kemp et al., 2013; Lindecke et al., 2006; Nakazawa et al., 1993; Tischmeyer and Grimm, 1999). Similarly, perirhinal infusion of an ODN against brain-derived neurotrophic factor (BDNF) during the memory delay interval produced impairment of familiarity discrimination, establishing that BDNF is necessary for recognition memory consolidation mechanisms. Interestingly, perirhinal expression of BDNF is increased after performance of an object recognition memory task (Griffin et al., 2009; Hopkins and Bucci, 2010; Hopkins et al., 2011; Muñoz et al., 2010) and there are changes in perirhinal neurotrophin-related Trk receptors (Callaghan and Kelly, 2013). Recognition memory is also impaired by a tyrosine kinase antagonist that blocks neurotrophin-related Trk receptors (Callaghan and Kelly, 2013). BDNF is a neurotrophin that has been shown to be involved in synaptic plasticity in other systems (Bekinschtein et al., 2008; Bramham and Messaoudi, 2005; Lu et al., 2008; Pang et al., 2004; Woo et al., 2005).

**Fig. 5:**
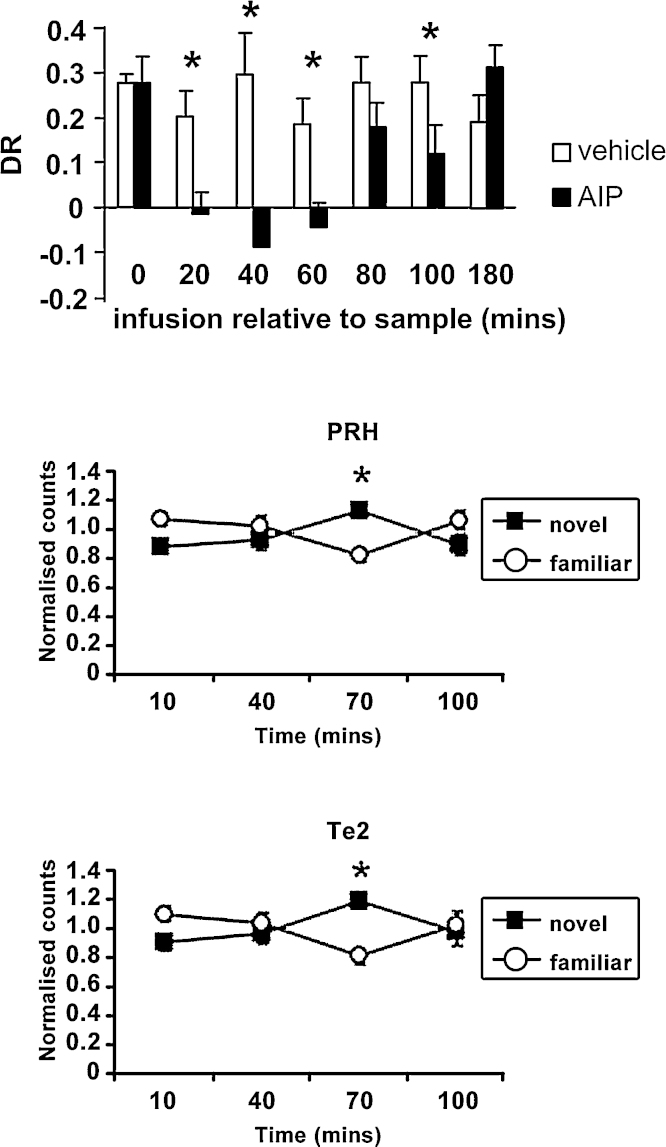
Phosphorylation of CamKII and familiarity discrimination. *Top panel*: Effect of perirhinal infusions of vehicle or the CAMKII inhibitor AIP (autocamtide-2-related inhibitory peptide) at differing times relative to acquisition on object recognition memory after a 24 h delay in the rat. Discrimination ratios (DR) were used as an index of memory performance (a DR of zero indicates no preference). *Time-points at which there was a significant effect of treatment. Amnesia followed infusions 20–60 min after acquisition. *Lower two panels*: Normalised counts of pCAMKIIa-stained neurons at different times after the paired viewing of novel and familiar stimuli for (*middle panel*) perirhinal region (PRH), (*bottom panel*) area Te2, *Significant differences between novel and familiar counts were found 70 min after viewing in both regions.

Adapted with permission from Figs. 3 and 4, Tinsley et al. (2009).

Perirhinal infusion of inhibitors of calcium–calmodulin-dependent kinases (CamKs) during a 24 h memory delay indicates that familiarity discrimination is dependent upon the phosphorylation of CamKII during a period 20–100 min after acquisition (Tinsley et al., 2009). Similarly, blocking activation of extracellular signal-regulated kinase (ERK) activation in perirhinal cortex after acquisition produces recognition memory impairment (Silingardi et al., 2011). Importantly, such inhibitors should not disrupt perirhinal transmission, so that perirhinal transmission of information to other areas should remain equivalent to that before the learning during the sample phase. In particular, perceptual processes that do not require learning-related changes should remain intact. Hence the familiarity discrimination impairment resulting from localised infusion of perirhinal cortex with inhitors of CamKII phosphorylation provides strong evidence that perirhinal cortex is indeed an information storage site (Fig. 5).

As mentioned above, association cortex (Te2) adjacent to perirhinal cortex is also involved in recognition memory processing: lesions result in impairment at long delays (Ho et al., 2011) and Fos differences are found using the paired viewing procedure (see for review: Aggleton et al., 2012). One of the main routes by which visual information reaches perirhinal cortex is via Te2 (Burwell and Amaral, 1998; Shi and Cassell, 1999; Sia and Bourne, 2008). However, information may also be fed back from perirhinal cortex to Te2: the necessary anatomical connections exist (Burwell and Amaral, 1998). Backwardly propagating signals from perirhinal cortex have been described during visual paired associate learning in the monkey (Naya et al., 2001, 2003). Importantly, in the rat, structural equation modelling of Fos changes during recognition memory performance in the bowtie maze indicates that there is indeed such information transfer (Aggleton et al., 2012; Albasser et al., 2010). Differential phosphorylation of CAMKII or CAMKK produced by viewing novel or familiar stimuli is found in Te2; however, this signal is lost following inhibition of such phosphorylation within perirhinal cortex, suggesting that the Te2 signal is dependent on perirhinal activity (Tinsley et al., 2009). In contrast, differential Fos expression produced in Te2 by viewing novel or familiar stimuli is not disrupted when Fos production is inhibited in perirhinal cortex (Seoane et al., 2012). These studies indicate a time window of >20 min to <3 h after acquisition for any backwardly propagating signal to pass from perirhinal cortex to Te2. As computational modelling suggests that the information storage capacity of perirhinal cortex could be sufficient for familiarity discrimination over a life-time, there are not clear theoretical grounds related to storage capacity for expecting information transfer out of perirhinal cortex into some alternative long-term store. Moreover, any backwardly propagating signal is insufficient to allow Te2 to maintain recognition memory after a 24 h delay if perirhinal processing is disrupted at the time of retrieval. Hence, although both Te2 and perirhinal cortex are necessary for long-term recognition memory, Te2 does not support such memory without perirhinal cortex.

## 6 Linking memory formation to electrophysiological measures of plasticity

**Fig. 6:**
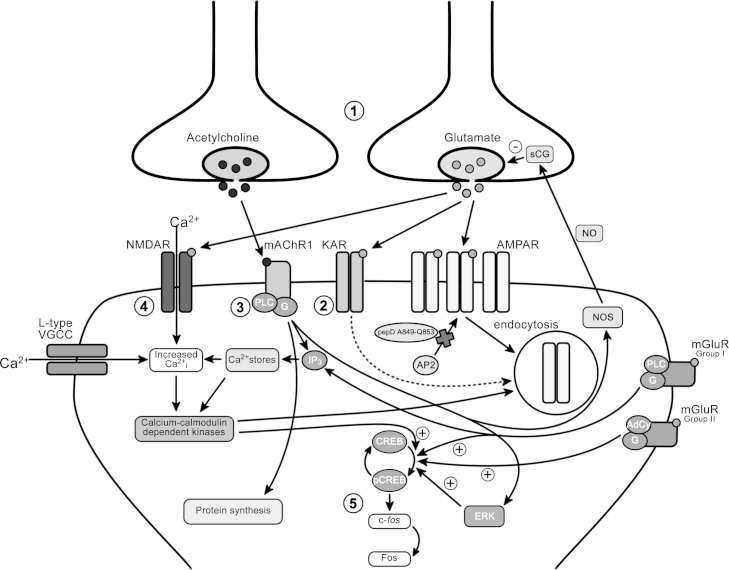
LTD pathways in perirhinal cortex. (1) Activation of glutamatergic and cholinergic afferents in perirhinal cortex, typically at 1–5 Hz, leads to release of neurotransmitter and activation of post-synaptic receptors. (2) Kainate receptor (KAR) activation is required for familiarity discrimination at short (≤20 min) but not longer delays. Although there are no currently known roles for kainate receptors in perirhinal plasticity, it is known that inhibition of AP2-dependent AMPA receptor endocytosis by pepD849-Q853 also impairs object recognition memory at short delays, thus suggesting that KARs are also involved in synaptic weakening processes. (3) Cholinergic modulation of perirhinal cortex is implicated in both learning and protein synthesis-dependent LTD. Muscarinic acetylcholine receptor 1 (mAChR1) activation leads to release of calcium from intracellular stores and subsequent activation of calcium-sensitive kinases, and additionally stimulates extracellular-signal related kinases (ERK) leading to phosphorylation of CREB and production of Fos protein. Muscarinic receptor activation also activates nitric oxide synthase (NOS), producing nitric oxide (NO) which can act as a retrograde signalling molecule, activating soluble guanylate cyclase which attenuates glutamate release. Block of mAChR1s during acquisition impairs object recognition memory at delays of up to 6 h, whilst inhibition of NOS impairs memory at a delay of 24 h, suggesting mechanisms other than mAChR1 may also stimulate NO production in perirhinal cortex. (4) Activation of L-type voltage gated calcium channels (VGCCs), mGluRs and GluN2B-containing NMDA receptors are all required for object recognition at a 24 h delay. Activation of these proteins leads to increases in intracellular calcium concentration and calcium–calmodulin dependent kinase (CamK) activation which is thought to phosphorylate AMPA receptors and facilitate their endocytosis. (5) CREB phosphorylation is required for object recognition memory at a 24 h delay and is increased by mGluR, mAChR1 and calcium–calmodulin dependent kinase (CamK) activation. Phosphorylated CREB stimulates transcription and is known to lead to production of Fos protein. Although these events are required for object recognition memory and LTD, it is currently unclear how these processes lead to synaptic weakening. It is however known that endocytosis of AMPA receptors by clathrin adaptor protein AP2 is required for both LTD and object recognition memory at delays of 5 min or 24 h.

Adapted with permission from Fig. 3, Banks et al. (2012).

As synaptic changes are hypothesised to be involved in the encoding of memory, and response changes have been recorded in perirhinal cortex in response to learning, it follows that pharmacological manipulations which impair memory should also impair synaptic plasticity if such plasticity mechanisms are responsible for memory storage. This relationship has been investigated using electrophysiological recordings of in vitro brain slice preparations. Synaptic enhancement (long-term potentiation: LTP) and synaptic weakening (long-term depression: LTD) can be produced in perirhinal cortical slices by appropriately patterned electrical stimulation (Liu and Bilkey, 1996; Ziakopoulos et al., 1999) (Fig. 6). For the most part those compounds which impair recognition memory have also been found to block induction or expression of synaptic plasticity in vitro, supporting the hypothesis that synaptic plasticity underlies recognition memory and informing us to some extent of the direction of plasticity affected by pharmacological intervention. However, one must be cautious not to use in vitro plasticity data as a proxy for recognition memory processes – the tone of afferent inputs to perirhinal cortical slices is significantly distorted. Furthermore, the stimulation patterns delivered to slices to induce activity-dependent synaptic plasticity are usually not consistent with those which occur in vivo, often stimulation lasting many seconds or minutes is applied to bring about synaptic changes which are thought to be expressed in a matter of milliseconds in behaving animals (Xiang and Brown, 1998), although exploration of novel object-place arrangements have been shown to affect induction of hippocampal plasticity in vivo using such protocols (Kemp and Manahan-Vaughan, 2004). Nevertheless, plasticity studies have proved useful tools in deciphering the molecular changes which may occur in the perirhinal cortex during object recognition memory formation. Electrophysiological studies of plasticity in chick IMM have similarly sought to make links to learning (e.g. Bradley et al., 1991a,b, 1992, 1999).

### 6.1 Links to plasticity induction mechanisms

Infusion of the non-subunit selective NMDA receptor antagonist AP5 into perirhinal cortex before the sample phase blocks familiarity discrimination at long, but not short delays (Barker et al., 2006b). Linking this phenomenon to a single plasticity process is problematic as all forms of perirhinal plasticity: LTD and its reversal, de-depression; LTP and its reversal, depotentiation (Liu and Bilkey, 1996; Massey et al., 2004; Ziakopoulos et al., 1999) are also inhibited by NMDA receptor inhibition.

NMDA receptors are tetramers which consist of two obligatory GluN1 subunits, and two GluN2 subunits of which there are four subtypes, GluN2A-D. In the forebrain GluN2A and GluN2B predominate and NMDA receptors are thought to primarily exist in either GluN2A- or GluN2B-containing species, or as triheteromeric receptors containing both GluN2A and GluN2B subunits. Investigation of plasticity in perirhinal cortex showed that selective inhibition of GluN2A-containing receptors with NVP-AAM077 prevented induction of both LTP and depotentiation, whilst, conversely, the GluN2B selective antagonist Ro 25-6981 blocked LTD only (Massey et al., 2004). This separation presented the opportunity to attempt to differentiate between plasticity processes involved in acquisition of recognition memory. However, selective inhibition of either only GluN2A-containing or GluN2B-containing receptors failed to produce an impairment in recognition memory at 24 h, although co-infusion of the two antagonists did produce amnesia (Barker et al., 2006b). The conclusion is that either more than one plasticity mechanism is able to support recognition memory, or that there are differences between learning triggers in vivo and plasticity triggers in vitro.

Several compounds exist which when infused into perirhinal cortex impair object recognition memory at long but not short delays and which also impair the induction of perirhinal cortex plasticity, particularly synaptic weakening: LTD or depotentiation. Simultaneous antagonism of both group I and II metabotropic glutamate receptors impaired acquisition of object recognition memory, but not its consolidation or retrieval (Barker et al., 2006a). In plasticity studies, antagonism of group I and II mGluRs did not impair LTP or depotentiation (Ziakopoulos et al., 1999), but group I mGluRs and group II mGluRs (under certain circumstances) were necessary for LTD induction (Cho and Bashir, 2002; Cho et al., 2000). Similarly, intraperirhinal infusion of verapamil (a blocker of L-type voltage-dependent calcium channels) before acquisition impaired object recognition memory at long, but not short delays and, like mGluR antagonists, blocked the induction of LTD and depotentiation, but not LTP (Seoane et al., 2009). These pharmacological interventions which cause recognition memory deficits and also inhibit induction of LTD and/or depotentiation, but not LTP, provide support for the idea that LTD-like synaptic weakening processes support recognition memory at long delays (Brown and Xiang, 1998). Nicotinic α7 receptor antagonism impairs familiarity discrimination at 24 h, but not 20 min delays (Tinsley et al., 2011) and nicotinic agonists enhance recognition memory at 72 h (Melichercik et al., 2012); however, as yet nicotinic effects on perirhinal plasticity are unknown. The benzodiazepine lorazepam (which enhances GABA-ergic inhibition) impairs recognition memory at both long and short delays and both LTP and LTD (Wan et al., 2004); this parallelism is consistent with the amnesia arising from impaired synaptic weakening processes, but does not supply strong support for the suggestion.

The cholinergic agonist carbachol induces LTD in perirhinal slices (Massey et al., 2001). Conversely, the muscarinic antagonist scopolamine blocks induction of activity-dependent LTD (Warburton et al., 2003). Perhaps the clearest link between learning and muscarinic-receptor plasticity comes from studies where the paired viewing procedure was used to expose one eye to highly familiar images and the other eye to many novel stimuli (both eyes were exposed to the same overall number of images). Brain slices were prepared from the two hemispheres. Plasticity was normal in the hemisphere which processed novel information, but in slices from the hemisphere processing familiar images both LTD and depotentiation were impaired while LTP was unaffected (Massey et al., 2008). The lack of any effect produced by viewing novel stimuli may be related to the potentially very high storage capacity of perirhinal cortex (Bogacz and Brown, 2003): the percentage of changed network synapses could be too small to measure. The change in plasticity produced by viewing familiar stimuli may reflect a change in plasticity thresholds from repeated learning about the familiar stimuli. The change could relate either to reconsolidation mechanisms (Nader et al., 2000) evoked when the previously seen stimuli were re-encountered, or to changes induced in the network when stimuli were viewed repeatedly (Woloszyn and Sheinberg, 2012). Recordings in monkeys indicate that there are increases in perirhinal neuronal responsiveness to stimuli seen many times (Holscher et al., 2003). Strikingly, plasticity was normal in the slices from the hemisphere processing familiar information when rats were given scopolamine to be active during the paired viewing procedure (Massey et al., 2008); the scopolamine would have blocked LTD-like processes and impaired memory acquisition (Warburton et al., 2003). However, further work is required to elucidate the particular mechanisms affected by the drug.

Although there are strong links between familiarity discrimination learning and LTD and depotentiation-like processes, a simple extrapolation of plasticity processes to learning (i.e. “LTD is sufficient for familiarity discrimination”) is not supported by all the available evidence. Whilst agonists of muscarinic receptors induce LTD, their antagonists only impair recognition memory at delays up to 3 h (Tinsley et al., 2011; Warburton et al., 2003). Likewise, GluN2B antagonist Ro 25-6981 does not impair learning (Barker et al., 2006b). Thus it appears that no single plasticity mechanism can be used to explain learning mechanisms underlying perirhinal-dependent familiarity discrimination. Indeed, the complexity is illustrated by the results of co-infusion of nicotinic and muscarinic antagonists into perirhinal cortex: whilst one compound blocked short-delay and the other long-delay recognition memory, when they were combined short-delay memory was spared, rather than the expected impairment at both delays (Tinsley et al., 2011).

Further evidence for the relative importance of LTP-like and LTD-like processes in familiarity discrimination has come from studies of retrograde messengers in perirhinal plasticity. Perirhinal LTP requires retrograde signalling via the CB1 endocannabinoid receptor; in contrast, LTD, either activity-dependent or carbachol induced, requires retrograde signalling via nitric oxide (Tamagnini et al., 2013). Local infusion of cannabinoid antagonists did not impair recognition memory at either long or short delays; however, blocking nitric oxide signalling produced a deficit at 24 h but not 20 min, again supporting the importance of LTD-like processes in recognition memory (Tamagnini et al., 2013).

Although NMDA receptor-dependent LTP has been described in perirhinal cortex, recently a form of NMDA receptor-independent plasticity has been found in a projection from the amygdala to perirhinal cortex (Perugini et al., 2012). Unlike LTP generated in perirhinal slices by stimulation of local cortical afferents (‘intracortical LTP’), this plasticity required activation of β-adrenoceptors and L-type voltage-dependent calcium channels (Perugini et al., 2012). The threshold for induction of intracortical LTP was reduced by co-activation of the amygdala pathway, giving rise to the suggestion that the amygdala may modulate perirhinal memory processes via heterosynaptic plasticity (Perugini et al., 2012). Bilateral lesions of the amygdala have been shown to impair familiarity discrimination, but not recollection, in a rewarded odour recognition memory task (Farovik et al., 2011). As a previous study utilising the same odour recognition memory task found that hippocampal lesions impaired recollection whilst sparing familiarity (Fortin et al., 2004), these data support the theory that the amygdala participates in processing of stimulus familiarity in an area other than hippocampus, almost certainly perirhinal cortex.

Structural elements may also influence learning and plasticity in perirhinal cortex. Familiarity discrimination was tested in two cohorts of mice with depleted perineuronal nets, in one cohort the gene Ctrl1, a link protein, was unconditionally knocked out, and in the other the perineuronal net was enzymatically degraded. Control mice could perform familiarity discrimination at delays up to only 3 h, but cohorts of mice with depleted perineuronal nets were able to discriminate at 48 h delays (Romberg et al., 2013). Both the transgenic and the enzymatically treated mice showed enhanced LTD in slices, possibly again implicating LTD-like processes in object recognition memory (Romberg et al., 2013). It should be noted that basal synaptic transmission was also enhanced in slices from animals with depleted perineuronal nets when compared to control animals; accordingly, enhanced memory might have been a result of enhanced transmission rather than enhanced storage (Romberg et al., 2013). In a further link between LTD and memory, in a mouse model of Alzheimer's disease, abnormally pronounced perirhinal LTD was linked to recognition memory deficits; both memory and LTD were returned to levels seen in control mice by the NMDA receptor antagonist memantine (Romberg et al., 2012; though see: Tamagnini et al., 2012).

### 6.2 Links to the expression and maintenance of plasticity

The evidence outlined above shows strong evidence that pharmacological agents that interfere with the induction of perirhinal plasticity also cause memory deficits, most commonly when they are given to be active during acquisition (and therefore also during the first stages of consolidation). It is possible, however, that these agents affect transmission from perirhinal cortex to other regions (e.g. hippocampus) in addition to disrupting storage processes in perirhinal cortex itself, thereby leaving open alternative explanations for the memory deficits they produce. To strengthen links between synaptic changes in perirhinal cortex and learning, investigations have attempted to disrupt plasticity processes by blocking plasticity transduction and expression mechanisms, with the aim of selectively impairing memory even when the operation of the recognition memory system during acquisition is normal.

Downstream of ion channels and neurotransmitter receptors, various intracellular signalling molecules which have been linked to plasticity, either in perirhinal cortex or other brain regions such as the hippocampus, have also proven to be involved in recognition memory processes. One such molecule, CamKII, is implicated in expression of LTP and LTD (Malenka and Nicoll, 1999; Mayford et al., 1995; Pi et al., 2010). In vitro inhibition of CamKII blocks the induction of LTP without affecting basal transmission (Cho et al., 2001; Malinow et al., 1989; Rodrigues et al., 2004). Hence the impairment of familiarity discrimination produced by infusion of CamKII inhibitors into perirhinal cortex is most likely to be due to impairment of consolidation mechanisms in that cortex (Tinsley et al., 2009): perceptual processes and transmission of information within the recognition memory system not requiring memory storage should be unaffected.

Extracellular signal related kinases (ERKs) have also been implicated in long-term memory and synaptic plasticity (Brambilla et al., 1997; Silingardi et al., 2011). Infusing ERK inhibitors into perirhinal cortex produced an object recognition memory deficit at long delays (Silingardi et al., 2011). RasGRF1 knockout mice, which show reduced ERK activation in response to neuronal activation, were found to have impaired familiarity discrimination at longer than 1 h delays, whilst ERK 1 knockout mice, in which ERK2 is more sensitive to neuronal activity, had better memory at long delays than wild type mice (Silingardi et al., 2011). These transgenic mice showed corresponding changes in the magnitude of in vitro synaptic plasticity: ERK1 knockout mice had greater LTP and LTD than controls, whilst plasticity was reduced in RasGRF1 knockouts.

Further downstream of CamKs and ERKs, CREB (cAMP responsive element-binding protein) is phosphorylated to pCREB which activates transcription of genes including c-*fos*. Viral transduction of perirhinal cortex with a dominant negative ligand of CREB, produced a deficit in long-delay recognition memory and a deficit in perirhinal LTP (Warburton et al., 2005); LTD was not studied. Furthermore, after such blocking of the CREB pathway there was no longer the normal novel-familiar difference in Fos counts in perirhinal cortex following the paired-viewing procedure (Warburton et al., 2005). As mentioned previously, viral delivery of antisense Fos oligonucleotide into perirhinal cortex impaired object recognition memory at delays ≥3 h (Seoane et al., 2012).

In an attempt to link plasticity and learning more closely, Griffiths and colleagues (2008) tested the hypothesis that perirhinal LTD mechanisms are involved in recognition memory by blocking the primary expression mechanism of LTD. Removal of AMPAR receptors from the post-synaptic density by endocytosis is thought to be the principal expression mechanism of LTD (Henley, 2003; Malenka and Bear, 2004; Massey and Bashir, 2007). The final stage is mediated by clathrin adaptor protein AP2 interacting with GluA2 subunits of AMPARs (Beattie et al., 2000; Carroll et al., 1999; Luscher et al., 1999; Luthi et al., 1999). Peptidergic blockade of this interaction using pepD849-Q853 blocked perirhinal LTD without effecting basal transmission or LTP, and viral transduction of perirhinal cortex that generated the blocking peptide produced recognition memory deficits whilst sparing object-in-place memory (Griffiths et al., 2008). That this approach does not alter basal AMPA receptor mediated transmission, nor that of GABA_A_ or NMDA receptors (Griffiths et al., 2008), means that transmission of information via perirhinal cortex is unaffected. As LTP is unaffected, the findings provide the strongest evidence to date that LTD-like mechanisms in the perirhinal cortex are necessary for object recognition memory, and that perirhinal cortex acts not only as a transmission region but also as a memory store.

A similar approach has been taken to alter the expression of LTP. One mechanism of increasing synaptic gain following LTP induction is insertion of AMPA receptors into he postsynaptic density (others include changes in the biophysical properties of AMPA receptors and changes in presynaptic transmitter release (Malenka and Bear, 2004)). Maintaining this newly increased number of AMPA receptors is thought to be an ongoing process which involves inhibition of an AMPA receptor endocytosis pathway affecting GluA2-containing receptors – a process that may be mediated by the atypical PKC isoform PKMζ (Sacktor, 2011). The activity of PKMζ can be blocked by PKMζ inhibitory peptide, ZIP (Yao et al., 2008). ZIP has been shown to reverse established LTP in both the hippocampus (Ling et al., 2002), and in perirhinal cortex (Panaccione et al., 2013), whilst sparing LTD (Panaccione et al., 2013). Furthermore, ZIP has been shown to produce retrograde amnesia when infused into a number of brain regions including amygdala and hippocampus (Sacktor, 2011). With respect to recognition memory, ZIP infusion had no effect on object recognition memory when infused into perirhinal cortex between 20 min and 3 h before acquisition (Outram et al., 2010), whereas ZIP abolished memory when given immediately after acquisition, or when given 24 h post-sample using a 48 h delay interval (Outram et al., 2010). As ZIP is known to reverse LTP in perirhinal slices (Panaccione et al., 2013), these effects are most easily explained by effects on plasticity mediated by actions on PKMζ. However, it is possible that an alternative mechanism is responsible for the memory loss. The memory deficits were thought to be due to inhibition of PKMζ by ZIP, and the consequent prevention of the blockade of AMPA receptor endocytosis, which thereby allowed any newly inserted AMPA receptors to be removed from strengthened synapses (Migues et al., 2010). However, the specificity of ZIP for PKMζ has recently come into doubt. It has been shown that ZIP can also inhibit another atypical PKC isoform, PKCλ/ι (Lee et al., 2013; Ren et al., 2013), which is involved in the induction of LTP (Ren et al., 2013). Furthermore, PKMζ knockout mice have been shown to be able to perform various forms of hippocampal dependent memory tasks, whilst showing normal hippocampal synaptic transmission and plasticity which, crucially, remains sensitive to reversal by ZIP (Volk et al., 2013). In a further transgenic study with PKMζ knockout mice, ZIP could still reverse cocaine-induced place preference when infused into nucleus accumbens (Lee et al., 2013). Whilst the mechanism of ZIP's effects are uncertain, it remains a useful tool as ZIP remains the only compound both to reverse established LTP in vitro and to induce retrograde amnesia in vitro. Nevertheless, the mechanism by means of which ZIP produces recognition memory impairment remains to be determined.

## 7 Summary

We have presented evidence to show that perirhinal cortex is necessary for single-trial learning related to familiarity discrimination for individual items. Furthermore, there is considerable evidence to show that not only is transmission of information via perirhinal cortex is necessary for object recognition memory, but that the perirhinal cortex is also an information store. This contention is strongly supported by the non-temporally graded retrograde amnesia following lesions of perirhinal cortex, and by pharmacological and molecular manipulations which may produce amnesia by affecting storage, consolidation or retrieval mechanisms within perirhinal cortex. In particular, there is evidence that memory may be impaired by interfering with plasticity consolidation and storage mechanisms within perirhinal cortex without affecting normal perirhinal transmission.

The hypothesis that memory storage within perirhinal cortex is mediated by changes in synaptic weight is supported by links between impairments in memory and in vitro synaptic plasticity in vitro, and by observations from in vivo electrophysiological recordings which show neuronal response changes to visual stimuli. Furthermore, there are consistent findings across numerous studies indicating that synaptic weakening remains a key mechanism underlying object recognition memory. Moreover, computational modelling has established the necessity of synaptic weakening to efficient network performance of familiarity discrimination and the potentially adequate storage capacity of such a system of the size of perirhinal cortex. However, evidence also suggests that a single synaptic plasticity process is not sufficient to sustain object recognition memory and questions remain regarding the temporal dynamics of and molecular mechanisms underlying acquisition, consolidation and storage processes. Furthermore, the processing of familiarity discrimination beyond single exposure learning for individual items (as primarily discussed in this review) is known to involve brain regions beyond the perirhinal cortex (including but not limited to hippocampus and medial prefrontal cortex). Even less is known of the underlying mechanisms involved in these additional regions involved in recognition memory processes.
